# Personalized rehabilitation approach for reaching movement using reinforcement learning

**DOI:** 10.1038/s41598-024-64514-6

**Published:** 2024-07-30

**Authors:** Avishag Deborah Pelosi, Navit Roth, Tal Yehoshua, Dorit Itah, Orit Braun Benyamin, Anat Dahan

**Affiliations:** 1 Mechanical Engineering Department, Braude College of Engineering, Karmiel, Snunit 51 St., 2161002 Karmiel, Israel; 2 Software Engineering Department, Braude College of Engineering, Karmiel, Snunit 51 St., 2161002 Karmiel, Israel; 3 Western Galilee Medical Center, P.O.B. 21, 2210001 Nahariya, Israel

**Keywords:** Adaptive rehabilitation, Virtual reality, Reinforcement learning, Reaching movement, Serious games, Rehabilitation, Computer science

## Abstract

Musculoskeletal disorders challenge significantly the performance of many daily life activities, thus impacting the quality of life. The efficiency of the traditional physical therapy programs is limited by ecological parameters such as intervention duration and frequency, number of caregivers, geographic accessibility, as well as by subjective factors such as patient’s motivation and perseverance in training. The implementation of VR rehabilitation systems may address these limitations, but the technology still needs to be improved and clinically validated. Furthermore, current applications generally lack flexibility and personalization. A VR rehabilitation game simulation is developed, which focuses on the upper-limb movement of reaching, an essential movement involved in numerous daily life activities. Its novelty consists in the integration of a machine learning algorithm, enabling highly adaptive and patient-customized therapeutic intervention. An immersive VR system for the rehabilitation of reaching movement using a bubble popping game is proposed. In the virtual space, the patient is presented with bubbles appearing at different locations and is asked to reach the bubble with the injured limb and pop it. The implementation of a Q-learning algorithm enables the game to adjust the location of the next bubble according to the performance of the patient, represented by his kinematic characteristics. Two test cases simulate the performance of the patient during a training program of 10 days/sessions, in order to validate the effectiveness of the algorithm, demonstrated by the spatial and temporal distribution of the bubbles in each evolving scenario. The results show that the algorithm learns the patient’s capabilities and successfully adapts to them, following the reward policy dictated by the therapist; moreover, the algorithm is highly responsive to kinematic features’ variation, while demanding a reasonable number of iterations. A novel approach for upper limb rehabilitation is presented, making use of immersive VR and reinforcement learning. The simulation suggests that the algorithm offers adaptive capabilities and high flexibility, needed in the comprehensive personalization of a rehabilitation process. Future work will demonstrate the concept in clinical trials.

## Introduction

Musculoskeletal disorders and diseases have a debilitating impact on the performance of daily life activities (ADL), negatively affecting the quality of more than 50% of the individuals aged more than 50 years old suffering from chronic conditions in the developed countries^1^. Traditional rehabilitation of motor capabilities and physical therapy involve the repetition of task-oriented movements according to personal disability, in one-on-one therapist-patient sessions. The rehabilitation program is generally customized to the unique needs of the individual patient and includes personal condition assessment, definition of goals and strategy to attain them, limited training period, pain management and measures of improvement^2,3^. The process is time-consuming, expensive, and yields practical and ecological limitations concerning the number of caregivers, therapy session duration, intervention frequency and geographic accessibility. Challenges in traditional physical therapy also include patient’s adherence to long-term or intensive rehabilitation regimen, which is a critical factor in achieving actual improvement and recovery in many impairment conditions^4^. The integration of assistive VR technologies addresses the disadvantages of conventional rehabilitation, proposing an alternative healthcare intervention system^5^.

Virtual Reality (VR) immerses the patient in a realistic or imaginary environment while stimulating the performance of cognitive and motor functions required in daily life. It elicits the user to interact with a virtual environment, generally in an engaging and playful way, thus encouraging repetitive and otherwise boring movements. Moreover, a virtual platform is highly flexible, as it enables home-training, adaptivity in the implementation of on-demand modifications, immediate feedback, therapist monitoring and maximal treatment customization^3,6,7^.

The use and benefits of VR in physical rehabilitation have been investigated for several pathological conditions^5^, including post-stroke injuries^8–11^, cerebral palsy^12^, trauma-related impairment^13^, multiple sclerosis^14^, Parkinson’s disease^15^, chronic joint pain and arthritis^16^, and more, showing positive rehabilitation outcomes.

This research proposes a VR rehabilitation program simulation, which focuses on the upper-limb movement of reaching for an object while standing, an essential movement involved in numerous daily life activities. Its novelty consists in the implementation of a machine learning algorithm, enabling highly adaptive and patient-customized therapeutic intervention.

Current VR simulation systems are used in physical treatment and evaluation metrics, but very few of them integrate machine learning; the majority of current VR solutions involve optimization algorithms and conditional statements to adapt the difficulty level of rehabilitation “serious games” (games that include a didactic purpose) according to performance^17–20^ and/or to supply feedback to the patient^21,22^. Chen et al.^23^ developed a virtual reality interactive game called Super Pop VR for children with cerebral palsy, where the player is supposed to pop as many bubbles as possible in a given time by moving his arms. The platform evaluates in real time reaching movement kinematic parameters such as movement time, path length, shoulder range of motion, elbow range of motion, and more. Game settings can be adjusted, like bubble location, size and shape, appearing and retaining times. Similar adaptive systems allow the assessment of upper limb functionality in people with tetraplegia^24^ or recovering from stroke^25^, with immersive^26^ or non-immersive platforms^27^. To quantify performance, kinematic variables are generally recorded and analysed, according to position/motion sensors and the challenge level can be configured accordingly^28^.

Nevertheless, the configuration is generally set a-priori according to periodic reports, and a fully customized and adaptive system with performance learning capabilities is still lacking.

Machine learning algorithms offer the possibility to upgrade VR rehabilitation serious games by adding higher degrees of interactivity and adaptivity; an artificial intelligent agent can continuously adapt the complexity of the task according to the reinforcement it receives from the patient’s performance and therapist’s requirements. Recent works have proposed the use of reinforcement learning in the fields of Computer-assisted cognitive training^29,30^ and assistive robotics^29,31^. The implementation of machine learning in VR upper-limb rehabilitation games focuses on diagnosis optimization, transfer of knowledge and monitoring progress throughout the rehabilitation process^32,33^. Few pioneering pilot studies make use of neural networks^34^ and Dyna-Q reinforcement learning algorithm^35^ to give real time feedback and modify game difficulty on-line. Barzilay and Wolf^34^ measure kinematics and muscle activity in a specific task to train a network, which can then generate new tasks for the individual trainee, based on his expected performance. Their immersive (HMD) system is designed for subjects with neuromotor disorders; the patient is asked to follow with his fingertip a planar trajectory, the adaptive system collects information from biometric equipment, produces an inverse model estimation of the measured performance and teaches, by mirroring, a more appropriate trajectory.

Tsiakas et al.^35^ use a desktop tele-rehabilitation system with multisensory data collection (including pain related facial expressions and speech) to give feedback and modify difficulty level of three exercises by implementing a Dyna-Q reinforcement learning (RL) algorithm. Contrarily to the model-free Q-learning methodology employed in our work, the Dyna-Q RL algorithm combines the model-free approach with offline simulation steps, aimed at requiring fewer real interactions although allowing some inaccurate actions.

Sekhavat^36^ makes use of Multiple-Periodic Reinforcement Learning for difficulty adjustment in separate periods; the desktop game consists of hitting the brightest ball among an arch of balls and measures the user’s performance (win/lose) with Kinect controllers. The algorithm adapts game properties such as speed, size of balls and distance between arches of ballsby means of an elaborated RL-based algorithm, including multiple periods and multiple states, as well as probabilistic two-level actions; the goal of the adaptation is to optimize user’s satisfaction accommodating for a win/lose balance, without considering the kinematic characteristics of patient’s movement or therapist’s rehabilitation recommendations.

It is important to note that all current works are feasibility studies, and their small number is still not enough to validate the feasibility and benefits of VR adaptive rehabilitation systems^37^; there is imperative need of methods, simulations and experiments in the field of adaptive, customized rehabilitation.

The current work proposes a novel immersive VR adaptive simulation approach, that tailors the VR space to the patient and adapts in real time a rehabilitation game, implementing a Q-learning algorithm and considering both patient’s biomechanical kinematics and therapeutic strategy. Compared to available RL algorithm-based similar works^35,36^, this proof-of-concept simulation offers an adaptive model-free machine learning approach for the rehabilitation of upper-limbs in ADL tasks; the adaptation is based on real performance only, and allows the customization of kinematic parameters and therapeutic recommendations.

## Methods

### Game description

**Figure 1:**
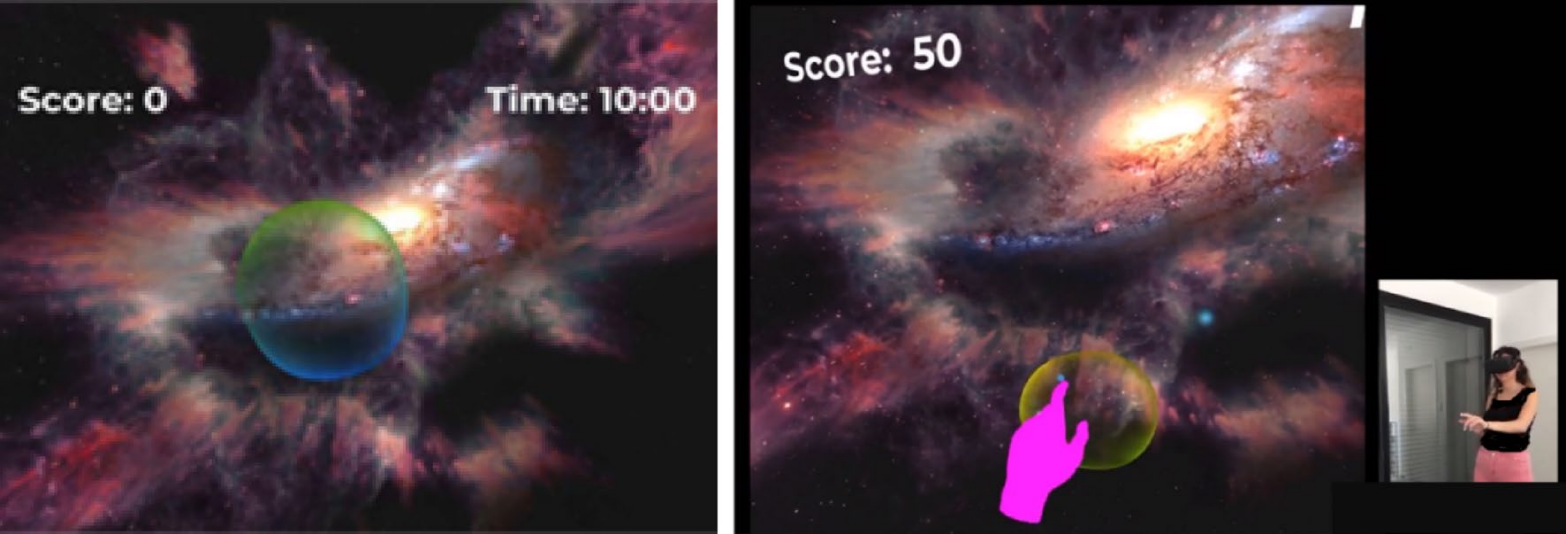
The virtual space as seen by the user. Left: a bubble appears, Right: the player’s hand represented by a virtual hand reaches and touches the bubble to pop it.

An immersive VR rehabilitation system for reaching movement using a bubble popping game is proposed. The game, implemented using Unity 3D Game Engine (Unity Technologies, v2021.1.9.f1), is conceived as a rehabilitation solution for all upper-limb disabilities, regardless of the pathology or injury involved. The system includes an Oculus Quest 2 (Meta Quest 2) Head-Mounted Display (HMD) with 4 embedded infrared cameras (Fig. 1). A customized application is employed to collect and store the controller’s position and orientation. This study reports the results of a proof-of-concept simulation, hence the Meta Quest 2 device is not actually used in a real setup.

In the virtual space, the patient is presented with bubbles appearing at different locations and he is asked to reach the bubble with his injured limb and to pop it by touch. The movement involves mainly the shoulder and elbow joints.

The game's space is divided into a 3D set of cubes (not visible to the player). At each step of the game, a bubble appears at a selected cube. The patient needs to physically reach and touch the bubble to explode it. If he doesn’t succeed, the bubble disappears after a fixed time, determined by the therapist’s setup. When a bubble is popped, it instantly disappears making a pop sound. In the virtual space the patient can see the elapsed time and his score, based on popping success/failure and kinematic measures. At the following step, a new bubble appears at another cube of the game space. Figure 1 shows the virtual space as it presents a bubble to the user. The position of the patient’s hand is represented by a hand in the virtual space. The dimensions and boundaries of the virtual space are defined according to the limits of reaching movements of a person without disabilities.

The use of the Reinforcement Learning approach and specifically Q-learning algorithm enables the customization of the rehabilitation serious game. The system allows personalized allocation of the bubbles as a function of the user’s changing physical capabilities; the game adjusts the location of the next bubble according to the previous performance of the patient. The individual user abilities represent the environment that the AI agent is to learn. The agent decides where to present the next bubble in order to get a maximum reward. The reward function models the policy of a personalized treatment, determined by therapist’s instructions. For example, it may give a high reward for bubble locations that are challenging for the patient; the algorithm will then learn the patient’s abilities and accordingly prefer to present bubbles in areas where the reward is high. This approach provides a dynamic personalization and adaptation of the VR rehabilitation process.

### Reinforcement Q-learning

Reinforcement learning is an area of machine learning that models how software agents should take actions in an environment in order to maximize a reward^38^: the selection of an action is achieved by a learning agent that interacts with the environment and tries to maximize a cumulative reward. The selected action may affect not only the immediate reward but also the next state of the environment and subsequent rewards. To obtain high rewards, a reinforcement learning agent will prefer an action that it has tried in the past and found to be effective in producing a reward. To make better action selections in the future, it also has to try actions that it has not selected before. At each time step *t,* the agent receives a representation of the environment’s state *S*_*t*_ and selects an action *A*_*t*_. At the following time step, according to the selected action, the agent receives a reward *R*_*t*+*1*_ and the environment’s state is *S*_*t*_ _+_ _1_.

Reinforcement learning systems have two decision-making approaches. The model-based method involves the use of a world predictive model, asking questions like “what will happen if I do *A*?”, to select the best action *A1* (such as Markov Decision Process). Conversely, the model-free approach skips the modelling step and directly learns a control policy.

Model-free reinforcement learning offers significant advantages in rehabilitation due to its adaptability, flexibility, and real-time feedback capabilities. Unlike model-based methods that require precise models of the environment or patient conditions, model-free approaches can adjust to varying patient responses and changing conditions without extensive retraining. This adaptability is crucial in rehabilitation, where progress can be nonlinear and unpredictable.

This work makes use of Q-learning algorithm, which is a popular reinforcement learning method, based on a model-free exploration and iterative learning of the environment. Q-learning is a natural candidate for model free RL, as it allows to model the 3D space by states and actions, transitioning between them. One key advantage of Q-learning is that it is an off-policy RL as compared to on policy—Sarse, Monte Carlo, temporal difference algorithms. Off-policy algorithms allow suboptimal decisions, therefore balancing exploration and exploitation effectively, which is suitable to dynamic rehabilitation settings.

The goal of Q-learning is to learn a policy that maximizes rewards. This is achieved by creating a table (Q-table) of scores (Q-values) for all possible scenarios. The table has rows representing states {*S*} and columns representing possible actions {*A*}. The Q-values are stored in the Q-table and are updated during training of the algorithm by the Q-function, which generates new optimal Q-values based on both present and expected future information. After a first initialization, the Q-table is continuously updated as the algorithm proceeds with its learning process, which ends as soon as the optimal expected value of the total reward is found.

After selecting an action during learning, the Q-value for a given state and action is replaced by a new value, evaluated as follows^39^:

1 $$Q^{new} \left( {S_{t} ,A_{t} } \right) \leftarrow \left( {1 - \alpha } \right) \cdot Q\left( {S_{t} ,A_{t} } \right) + \alpha \cdot \left[ {R_{t} + \gamma \cdot max\left( {Q\left( {S_{t + 1} ,A} \right)} \right)} \right]$$

The algorithm replaces the current Q-value, $Q\left( {S_{t} ,A_{t} } \right)$ with a new one, ${Q}^{new}\left({S}_{t},{A}_{t}\right)$ for the state ${S}_{t}$ and the action ${A}_{t}$, by calculating a weighted average of old and new information, where

- $\alpha$ is the learning rate that controls how much of the difference between previous Q-value and newly proposed Q-value is considered. A factor of 0 means the agent does not learn at all and a factor of 1 makes the agent consider only the most recent information.
- *R*_*t*_ is the reward for taking action ${A}_{t}$ at state ${S}_{t}$,
- $max\left(Q\left({S}_{t+1},A\right)\right)$ is the maximal expected future reward given the new state ${S}_{t+1}$ and all possible actions at the new state $A$.
- $\gamma$ is the discount rate that determines the present value and the importance given to future rewards. This is a value between 0 and 1. As γ is close to 0 the agent is concerned only with maximizing immediate rewards, whereas a γ value of 1 leads the agent to consider only long-term rewards.

The new Q-value then includes two parts weighted by the learning rate: the old value and the new learned value, which is the sum of the immediate reward and a discounted estimate of the optimal future value.

For the algorithm to explore new actions, other than the ones with maximum reward, an exploration rate $0<\varepsilon <1$ is adopted, such that the algorithm selects a random action a pre-determined percentage of the times:

2 $$A_{t} = \left\{ {\begin{array}{*{20}l} {maxQt\left( a \right)} \hfill & {with \,\,probability \, \epsilon } \hfill \\ {random\left( a \right)} \hfill & {with\,\, probability\, \left( {1 - \varepsilon } \right)} \hfill \\ \end{array} } \right.$$

The action ${A}_{t}$ is chosen either randomly with a probability of $\varepsilon$, or according to the best-scored action in Q-table with a probability of $\left(1-\varepsilon \right)$. This approach is called epsilon-greedy Q-learning^40^ and provides a balance between exploitation and exploration by random choice.

### Implementation of the Q-learning algorithm

In the game, the patient is presented with bubbles in a 3D space which he must reach and pop. At each round a bubble appears at a different location. To translate the problem of how to present the bubbles in the most important locations as a reinforcement learning problem, the following representation is used: the player represents the environment, and the state is the location of the bubble. The goal of the agent is to present bubbles in locations that will result in maximal reward.

Each cube in the space is given an initial reward value of zero. During the game, the algorithm learns the patient’s physical reaching abilities and changes the reward value accordingly (Eq. 1). As the actual ability of a patient is probably not symmetrical in all directions, different bubble locations will result in different rewards.

The agent learns to choose the next bubble location (the cube location) that maximizes the total reward value.

The reinforcement problem is modelled as follows:

- State (S)—{XYZ | X, Y, and Z describe the row, column, and depth of a cube in the space correspondingly}
- Action (A)—{Up, Down, Right, Left, Forward, Backward}
- Q-table—A 2D table that allows to calculate the maximum expected future reward for each state and each action. This is represented in a 2D table where every cell contains the value of Q-function Q(S, A), describing the Q-value of every possible action for each given state. The rows and the columns in the table represent the states and the possible actions, respectively, from each state.

For each state all the columns representing actions that lead to states within the game boundaries are initialized to zero, otherwise the values are initialized to − 100 (for example from the top row the action representing UP will be − 100).

At each step, the bubble appears at a certain location. That is the state of the environment. According to the performance of the patient at that stage the Q-table is updated, and the best action to take from that stage is selected. According to the selected action a bubble is selected in the next location.

The therapist can define a training strategy that can be translated to the reward function. The reward links the presentation of bubbles and the kinematic performance of the patient. There are two aspects to take into consideration: does the patient manage to pop the bubble? What are the kinematic characteristics of his movement towards the bubble?

Performance is thus represented by three cases:

- The patient doesn’t pop the bubble,
- The patient easily pops the bubble,
- The patient pops the bubble with difficulty—the movement is slow, unsmooth etc.

The training strategy is determined by the therapist, given that the higher the reward, the higher the number of bubbles presented in the location.

For the sake of the present simulation the following strategy is adopted: the therapist wishes to encourage practice at the limits of the patient’s range of motion (ROM) where there is a difficulty in movement, to display a small number of bubbles where movement is easy and display some bubbles where the bubbles are still unreachable:

- When the patient does not reach/pop the bubble, the given reward value is low: fewer bubbles are then presented in the relevant zone so that the patient will have the opportunity to try again without being frustrated or physically injured.
- When the patient pops the bubble “easily”, the reward value is given a low negative value. This means that this is not the zone to be trained and there is less interest in presenting bubbles in the zone.
- When the patient reaches the bubble “with difficulty”, the reward is high, so that more bubbles are displayed in the encouraged practice zone.

The difficulty of the patient is represented by the kinematic characteristics of his movement: they may address pathlength (*m1*), smoothness (*m2*), time duration from bubble appearance to pop (*m3*) and maximal speed (*m4*). In future experimental work, these parameters can be calculated from the data collected by the VR tracking system. In real game conditions, a VR headset including hand controllers with built-in cameras and sensors can supply hand tracking and identify the location of body and hands in the virtual space in relation to the headset.

The VR system can track movements without any additional external sensor, thus allowing to measure hand position and to evaluate the kinematic parameters *m1–m4.* In the present simulation, they are given normalized values ranging from 0 to 1 and a kinematics score *K* is calculated by averaging the kinematic characteristics:

3 $$K = \frac{1}{4} \cdot \left( {m1 + m2 + m3 + m4} \right)$$

In real trials a different weight may be determined for each kinematic parameter, taking into consideration the specific characteristics of the pathology involved. The kinematic parameters are normalized in order to reflect adequately the difficulty in performing the reaching task. For example, a longer pathlength could erroneously represent a distant bubble, instead of depicting a longer path that the patient performs because of his physical disability. Therefore, normalized pathlength *m1* takes into account the shortest pathlength to the bubble, so that a normalized longer distance represents an actual longer path to the bubble. Similarly, normalization of path duration, *m3*, should include information of an average time to target based on an average movement velocity, measured in current or former states.

The reward function $R_{t}$ for the conditions and adopted strategy cited above is calculated as follows:

4 $$\begin{array}{*{20}l} {(a)\, if\,\; pop = 0} \hfill & {R_{t} = 0.2} \hfill \\ {(b)\, if\,\; pop = 1 \,\&\, K \ge 0.8} \hfill & {R_{t} = - \;0.3} \hfill \\ {(c)\, if\,\; pop = 1 \,\& \,K < 0.8} \hfill & {R_{t} = 1 - 0.5 \cdot K} \hfill \\ \end{array}$$

It is important to note that the patient’s performance may change throughout the treatment. Some abilities may improve (due to practice and recovery) while others might deteriorate. Accordingly, the reward given when reaching a certain bubble may change. The Q-table is updated at each iteration according to the pseudo-code in Table 1, and thus reflects changes in patient’s performance.

**Table 1:**
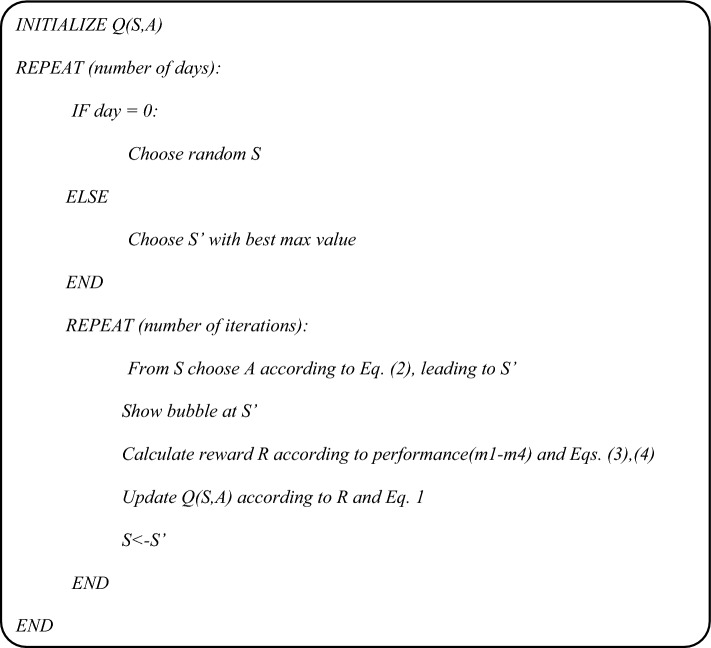
The pseudo-code of the Q-learning algorithm used in this study.

### Parameters of the algorithm

The use of the algorithm requires the selection of the parameters in Eq. (1):

- $\alpha$, the learning rate, is defined as *0.4*. This value balances previous with current performance allowing to learn without being too influenced from a single round.
- $\gamma$, the discount factor, is defined as *1*, to allow maximal influence of future rewards. This encourages the display of bubbles at locations with less reward that may lead to locations with higher reward.
- $\varepsilon$, the exploration rate defines the rate of random choice in the evaluation of the new Q-value, ${Q}^{new}\left({S}_{t},{A}_{t}\right)$ in Eq. (1). An epsilon-greedy approach allows to balance random exploration of random choices and exploitation of choices with maximal reward. At the beginning of the learning process the agent has no information about the capabilities of the user, therefore random action selection allows the algorithm to explore the environment and to discover new areas where there are motor difficulties. As the training progresses, we want to rely more on what the algorithm has learnt about the patient. We therefore use a dynamically decreasing epsilon-greedy approach as proposed by Wang et al.^41^ and Even-Dar and Mansour^42^. The following equation is used in this study:
  5 $${\varepsilon }\;{ = }\;\max \left( {\min \left( {\frac{E}{Total\, \,actions*Total \,\,reward},0.5} \right),0.3} \right) with \,E = 0.9$$

Accordingly, the exploration rate in the first sessions is high, i.e.

$\varepsilon =0.5$ and lowers to $\varepsilon =0.3$ in later sessions.

- *Episodes*—the number of VR sessions in this simulation is defined as *10 days.*
- *Iterations*—the number of bubbles presented for the patient to pop each day is *400*. A comparative study also considers the following values: *iterations* = *50, 400, 1000.*

### The simulation

**Figure 2:**
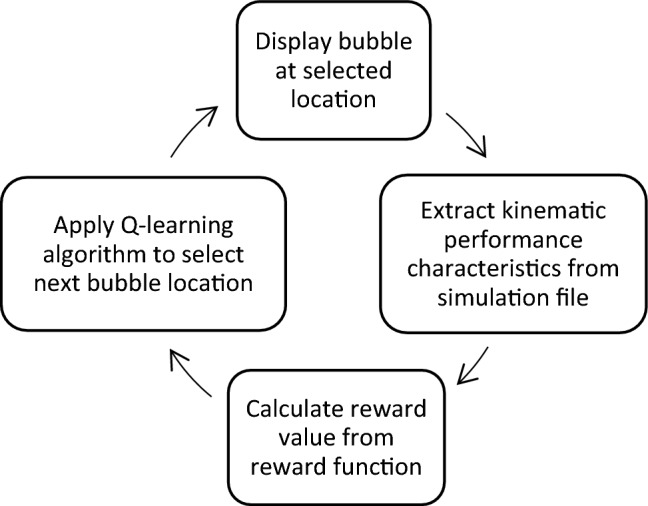
Flowchart describing testing of the algorithm via simulated kinematic properties.

In order to validate the algorithm, a simulation is presented in this paper as a preliminary stage before conducting clinical trials. The simulation flowchart is shown in Fig. 2: as soon as a bubble is displayed, kinematic characteristics featuring a test case are collected from a data file. The reward value is then calculated according to Eq. (4) and the Q-learning algorithm is applied to select the location of the next bubble as shown in Table 2.

**Table 2:** The kinematic characteristics of test case 1.

Days	Zone	Description	m1 course length	m2 trajectory smoothness	m3 time from bubble appearance to pop	m4 maximal speed
1–3	1	Can’t reach, can’t pop	0	0	0	0
	2	Pops with difficulty, stiff and slow movements	0.3	0.5	0.7	0.6
	3	Pops with ease	0.8–0.9	0.8–0.9	0.8–0.9	0.8–0.9
4–6	1	Pops with difficulty, stiff and slow movements	0.4	0.4	0.4	0.4
	2	Pops, quality of movements improves	0.6	0.8	0.8	0.9
	3	Pops with ease	0.8–0.9	0.8–0.9	0.8–0.9	0.8–0.9
7–10	1	Pops with difficulty, stiff and slow movements	0.4	0.4	0.4	0.4
	2	Pops with difficulty, stiff and slow movements	0.6	0.6	0.6	0.6
	3	Pops with ease	0.8–0.9	0.8–0.9	0.8–0.9	0.8–0.9

The VR simulation space is defined as a three-dimensional rectangular box, divided into 8 × 8 × 4 units: 8 cells in the X and Y axes, describing horizontal (right-left) and vertical (up-down) movements, respectively, and 4 cells in the Z axis, the movement’s depth (close-far).

**Figure 3:**
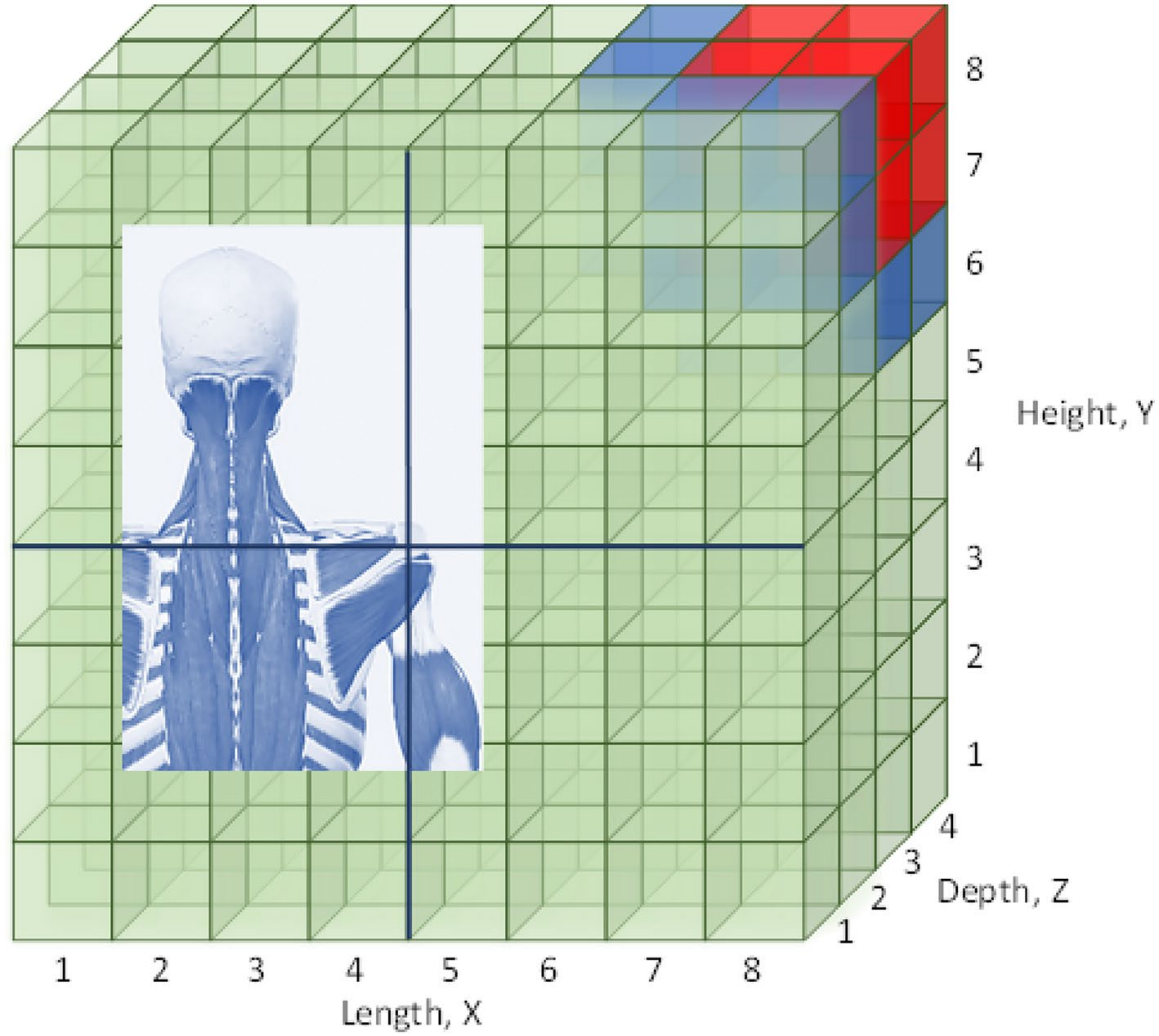
The simulation space, divided into three zones of varying performance: zone 1 (red) is the challenging target area, zone 2 (blue) around the target is reachable with difficulty, zone 3 (green) is the space with ease of movement.

The dimensions and the boundaries of the space are customized to each specific patient according to his height: anthropometrics measurements enable to relate individual’s height with his arm’s length^43^. The patient’s shoulder joint is located at location (*X,Y,Z*) = (*4,4,0*) as shown in Fig. 3.

The space is divided into three areas: the target area (zone 1), the zone around the target (zone 2) and the remaining space (zone 3). These zones represent locations of varying performance: the patient reaches differently in every zone (with varying degrees of difficulty/ease), thus presenting kinematic characteristics appropriate to each zone and scenario. A matrix of kinematic characteristics (*m1*, *m2*, *m3*, *m4*) changing with time and location simulates the patient’s performance and is used to calculate the reward.

The simulation addresses the everyday activity of reaching objects placed on a shelf in the right-upper corner of the patient’s kitchen/bathroom/closet. The patient has difficulties in reaching the shelf in the right upper corner of the VR space, depicted as zone 1 in red in Fig. 3.

The simulation provides a treatment tailored to the dynamic physical state of the user, defining kinematic characteristics that are changing in time during an estimated 10 sessions/days program of VR therapy.

Two test cases are considered, to assess the validity of the proposed Q-learning approach. To simplify the simulation and the understanding of its results, the course of the therapeutic program is divided in 3 periods of time with different kinematic performance: days 1–3, days 4–6 and days 7–10. In zones 1 and 2, that include relatively few spatial units (4 and 12, respectively), constant kinematic characteristics are adopted, whereas in the much larger zone 3 (240 units) a random variation in user’s high and low performance accounts for more realistic spatial kinetic differences: good performance is represented by random values of *K* values between 0.8 and 0.9 (*K* is defined in Eq. (3)), and low kinematic scores are evaluated as 30% of the good performance values (random *K* values between 0.24 and 0.27).

#### Test case 1

John comes to VR rehabilitation after a stroke. He suffers from muscle weakness and stiffness of his right arm and is advised occupational therapy to regain functionality in his daily activities. In the first three sessions (days 1–3) he cannot reach the right upper corner of the space (zone 1), he manages to pop bubbles in zone 2 with difficulty and easily reaches the bubbles in zone 3. In zone 2, his movements are relatively stiff and slow, resulting in low kinematic scores. In the next 3 sessions (days 4–6) his condition improves: he does succeed in popping bubbles in the target zone 1 with difficulty, the quality of his movements improve in zone 2 and easily reaches the bubbles in zone 3. In the last 4 VR sessions (days 7–10), John experiences fatigue that results in a minor slow-down in his recovery process. He pops bubbles in zone 1 with difficulty, his kinematic scores slightly decrease in zone 2, and he performs well as usual in zone 3.

The kinematic parameters corresponding to test case 1 are shown in Table 2: normalized values of *m1* (course length), *m2* (trajectory smoothness), *m3* (time duration from bubble appearance to pop) and *m4* (maximal speed) are evaluated in the different stages of the case study.

#### Test case 2

Mary suffers from a frozen shoulder. The range of motion of her right arm is impaired and she struggles with clothing and raising her hand to reach the right upper corner of her living space. In the first three sessions of her VR rehabilitation (days 1–3) she cannot reach target zone 1. She manages to pop bubbles in zone 2 with difficulty and pain but she can reach bubbles in zone 3 with ease. However, the training results in an increase in the intensity of the pain that she experiences, so that in the following 3 days (days 4–6) neither of zones 1 or 2 can be reached and in zone 3 bubbles are popped but movement is painful and difficult. In the last 4 sessions, Mary’s physical condition improves, and her kinematic scores are similar to those of days 1–3. The kinematic parameters corresponding to test case 2 are shown in Table 3.

**Table 3:** The kinematic characteristics of test case 2.

Days	Zone	Description	m1 course length	m2 trajectory smoothness	m3 time from bubble appearance to pop	m4 maximal speed
1–3	1	Can’t reach, can’t pop	0	0	0	0
	2	Pops with difficulty, stiff and painful movements	0.3	0.5	0.7	0.6
	3	Pops with ease	0.8–0.9	0.8–0.9	0.8–0.9	0.8–0.9
4–6	1	Can’t reach, can’t pop	0	0	0	0
	2	Can’t reach, can’t pop	0	0	0	0
	3	Pops with difficulty, stiff and painful movements	0.24–0.27	0.24–0.27	0.24–0.27	0.24–0.27
7–10	1	Can’t reach, can’t pop	0	0	0	0
	2	Pops with difficulty, stiff and painful movements	0.3	0.5	0.7	0.6
	3	Pops with ease	0.8–0.9	0.8–0.9	0.8–0.9	0.8–0.9

## Results

### Test case 1

Table 4 summarizes the results for test case 1. These include the number of bubbles, the number of bubbles divided by zone size and the sum of the rewards in each zone and training day.

**Table 4:** Results of test case 1: number of bubbles, number of bubbles/zone size and sum of rewards in every zone and training day.

	Result	Day									
		Day 1	Day 2	Day 3	Day 4	Day 5	Day 6	Day 7	Day 8	Day 9	Day10
Zone 1	Number of bubbles	43	26	13	205	331	337	317	353	354	351
	Number of bubbles/zone size	10.75	6.50	3.25	51.25	82.75	84.25	79.25	88.25	88.50	87.75
	Sum of rewards	8.60	5.20	2.60	164.00	264.80	269.60	253.60	282.40	283.20	280.80
Zone 2	Number of bubbles	172	317	331	165	61	46	67	45	40	39
	Number of bubbles/zone size	14.33	26.42	27.58	13.75	5.08	3.83	5.58	3.75	3.33	3.25
	Sum of rewards	126.85	233.79	244.11	101.06	37.36	28.18	46.90	31.50	28.00	27.30
Zone 3	Number of bubbles	185	57	56	30	8	17	16	2	6	10
	Number of bubbles/zone size	0.77	0.24	0.23	0.13	0.03	0.07	0.07	0.01	0.03	0.04
	Sum of rewards	− 55.50	− 17.10	− 16.80	− 9.00	− 2.40	− 5.10	− 4.80	− 0.60	− 1.80	− 3.00

According to the adopted treatment strategy, in the first three days the reward is the highest in zone 2, where John pops the bubbles with difficulties, whereas in zones 1 and 3 the reward should be low and negative, respectively. On day 1 the algorithm displays 43 bubbles in zone 1, 172 in zone 2 and 185 bubbles in zone 3. During the first three days the number of bubbles increases in zone 2 and decreases in zones 1, 3 as expected. The number of bubbles is divided by zone size, in order to take into account the significant difference between the zones: zone 1 includes 4 cubic units, zone 2 counts 12 units and zone 3 includes 240 units. Consequently, in zone 3 the number of bubbles divided by zone size is very low (< 1), showing very sparse bubble appearance in this large zone.

In the next three days (days 4–6), John’s performance improves in both zones 1 and 2: the number of bubbles in zone 1 immediately rises significantly, showing that the algorithm adapts quickly to the changing environment (the patient). The interest in zones 2 and 3 decreases, as John’s kinematic parameters there improve. In the last four days (days 7–10) John’s kinematic performance decreases in zone 2 from K = 0.775 (days 4–6) to K = 0.6. This change may seem minor but the algorithm responds with an initial increase in the number of bubbles in zone 2 (from 46 on day 6 to 67 on day 7) and a bubble number decrease in zone 1 (from 337 on day 6 to 317 on day 7), followed by a stabilization of the number of bubbles in all zones, with a consistent increase tendency in zone 1 and decrease in zones 2, 3. On day 10, 371 bubbles are presented in zone 1, 39 in zone 2 and 10 in zone 3, the bubble number divided by zone size is 87.75 for zone 1, 3.25 for zone 2 and 0.04 for zone 3.

**Figure 4:**
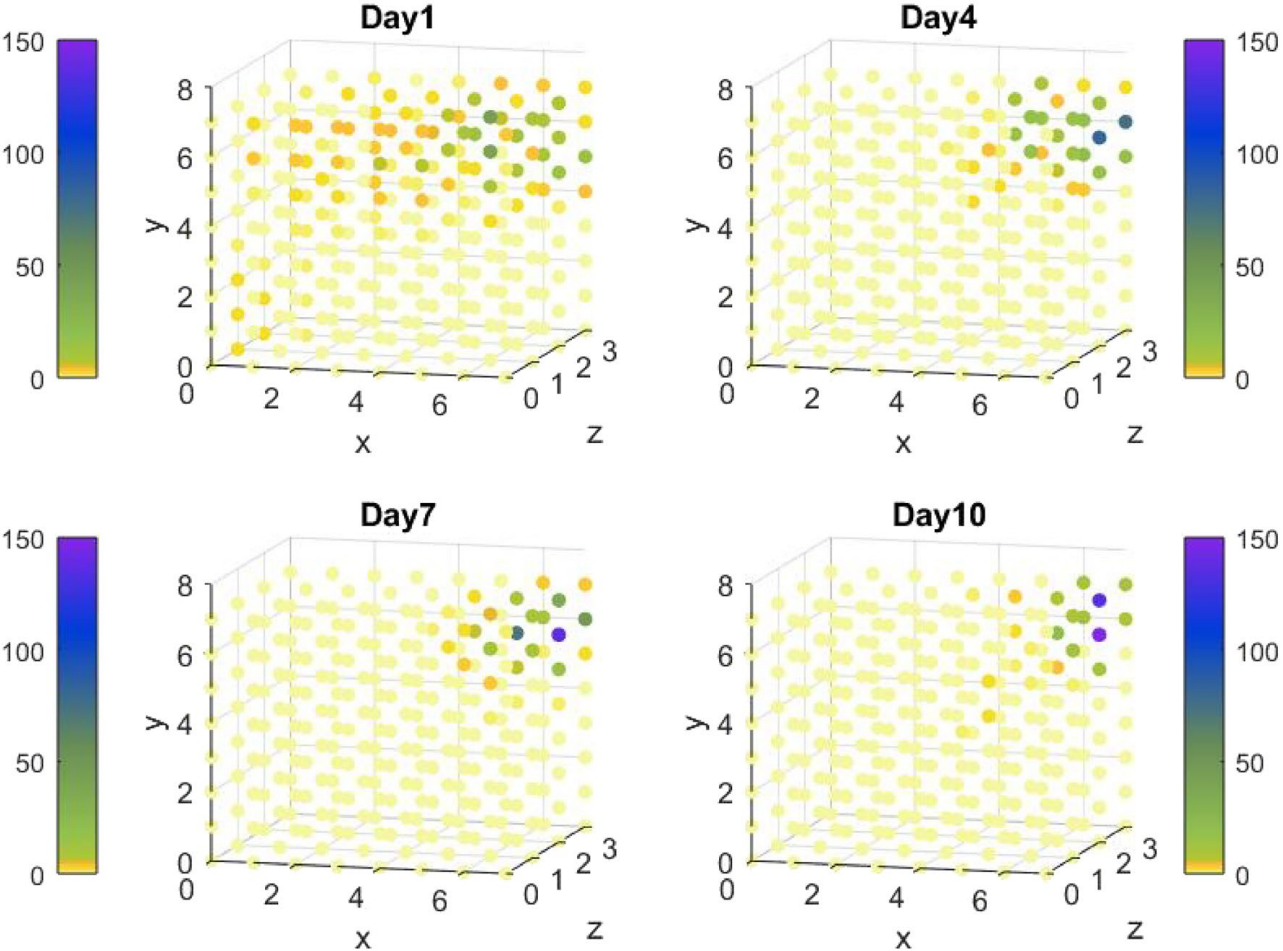
The number of bubbles appearing in the VR space on days 1,4,7 and 10 for test case 1.

Figure 4 shows graphically the number of bubbles appearing in the VR space on days 1, 4, 7, 10 for test case 1. The figure clearly shows a gradual increase in the number of bubbles in the upper right far corner of the space from day 1 to day 10.

**Figure 5:**
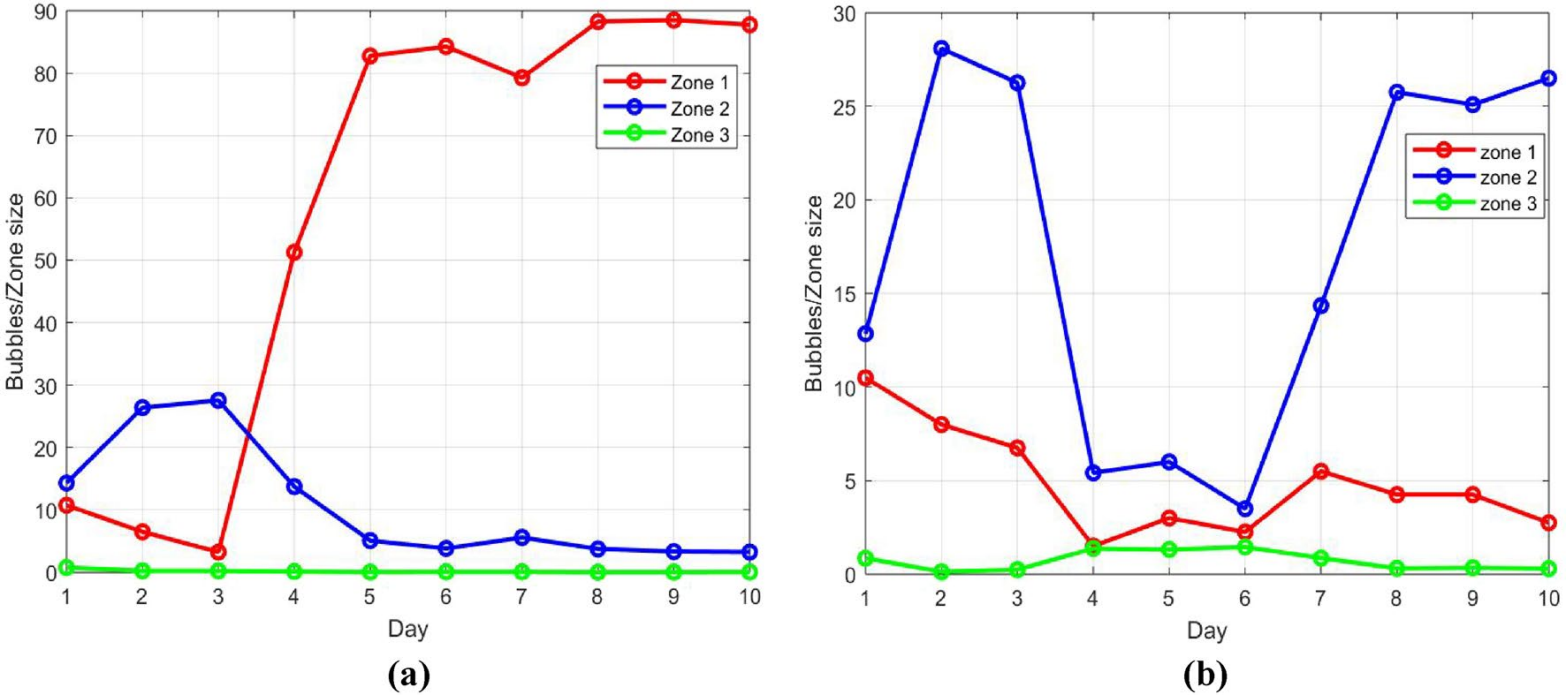
The number of bubbles divided by zone size for each zone and day in (**a**) test case 1, (**b**) test case 2.

In Fig. 5 the number of bubbles divided by zone size is plotted for each zone and day, for test case 1 (Fig. 5a) and test case 2 (Fig. 5b); the figure enables the visualization of bubble number variation in each zone with time. In test case 1 (Fig. 5a), the number of bubbles divided by zone size is so small in zone 3 that its variation is unnoticeable graphically. The main players in this test case are the bubbles in zone 1 and zone 2; the number of bubbles divided by zone size shows opposite line trends in zones 1 and 2, dictated by the reward policy and case scenario. From day 4 both zones converge to their final value with a significantly higher number of bubbles in zone 1, the target area. The adaptivity of the algorithm is demonstrated in its response to changes on days 4 and 7.

**Figure 6:**
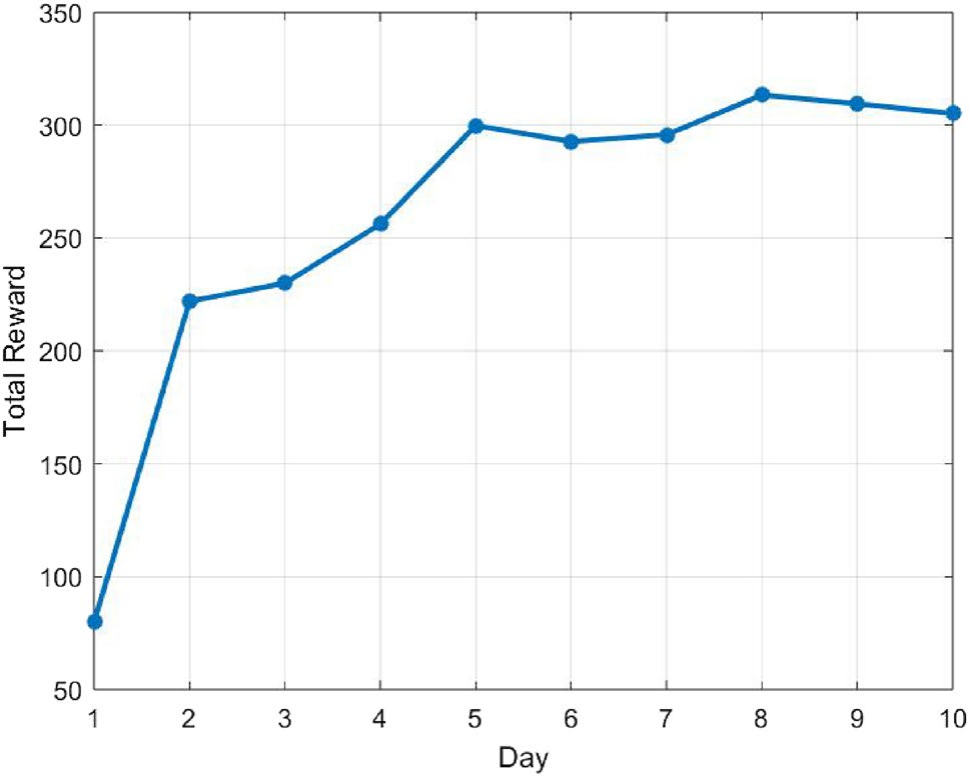
The total reward per day in test case 1.

Another indication of the algorithm’s learning process is the reward achieved. Table 4 shows the sum of rewards obtained in each zone and day. The sum of rewards in zone 2 is higher than in the other zones in the first three days, when John can’t pop bubbles in zone 1 and is encouraged to train in zone 2. Later on, when John can pop in zone 1, the training focus moves to this zone, with the highest sum of rewards. These results are consistent of course with the variation in the number of bubbles. In Fig. 6 the total reward, i.e., the sum of rewards in all zones, is displayed for each day. It is shown that the algorithm learns the patient’s abilities and therefore manages to get higher rewards as the training progresses. The total reward converges as required with this number of iterations (400).

### Test case 2

Table 5 summarizes the results of test case 2. In the first three days Mary cannot pop bubbles in zone 1, therefore the reward function defines a higher reward for zone 2 than for zone 1. In the following four days Mary’s condition worsens, she cannot reach zone 2 and has difficulties in reaching zone 3; in the last four days her abilities return to their initial values.

**Table 5:** Results of test case 2: number of bubbles, number of bubbles/zone size and sum of rewards in every zone and training day.

	Result	Day									
		Day 1	Day 2	Day 3	Day 4	Day 5	Day 6	Day 7	Day 8	Day 9	Day10
Zone 1	Number of bubbles	42	32	27	6	12	9	22	17	17	11
	Number of bubbles/zone size	10.50	8.00	6.75	1.50	3.00	2.25	5.50	4.25	4.25	2.75
	Sum of rewards	8.40	6.40	5.40	1.20	2.40	1.80	4.40	3.40	3.40	2.20
Zone 2	Number of bubbles	154	337	315	65	72	42	172	309	301	318
	Number of bubbles/zone size	12.83	28.08	26.25	5.42	6.00	3.50	14.33	25.75	25.08	26.50
	Sum of rewards	113.58	248.54	232.31	13.00	14.40	8.40	126.85	227.89	221.99	234.53
Zone 3	Number of bubbles	204	31	58	329	316	349	206	74	82	71
	Number of bubbles/zone size	0.85	0.13	0.24	1.37	1.32	1.45	0.86	0.31	0.34	0.30
	Sum of rewards	− 61.20	− 9.30	− 17.40	287.06	275.11	304.13	− 61.80	− 22.20	− 24.60	− 21.30

It is interesting to note that although John and Mary start with identical kinematic scores, on day 1 the number of bubbles displayed by the algorithm is not identical for both cases in all zones. This is due to the fact that the exploration rate parameter is high in the first days of training ($\varepsilon =0.5$), ensuring that the agent acts with a “healthy” degree of randomness. On the second and third days of learning, the algorithm converges quickly, displaying similar number of bubbles in both cases.

As shown in Table 5, in the first and last days the largest number of bubbles is displayed in Zone 2 (315 bubbles on day 3, 318 bubbles on day 10) and in days 4–6 in zone 3 (329 on day 4, 349 on day 6). This is consistent with the fact that Mary can pop bubbles in zone 2 at the beginning and the end of her rehabilitation, therefore the algorithm displays more bubbles in this zone. In days 4–6 her physical condition worsens and zone 2, together with zone 1, are not reachable, resulting in the algorithm’s adaptation presenting bubbles mainly in zone 3.

**Figure 7:**
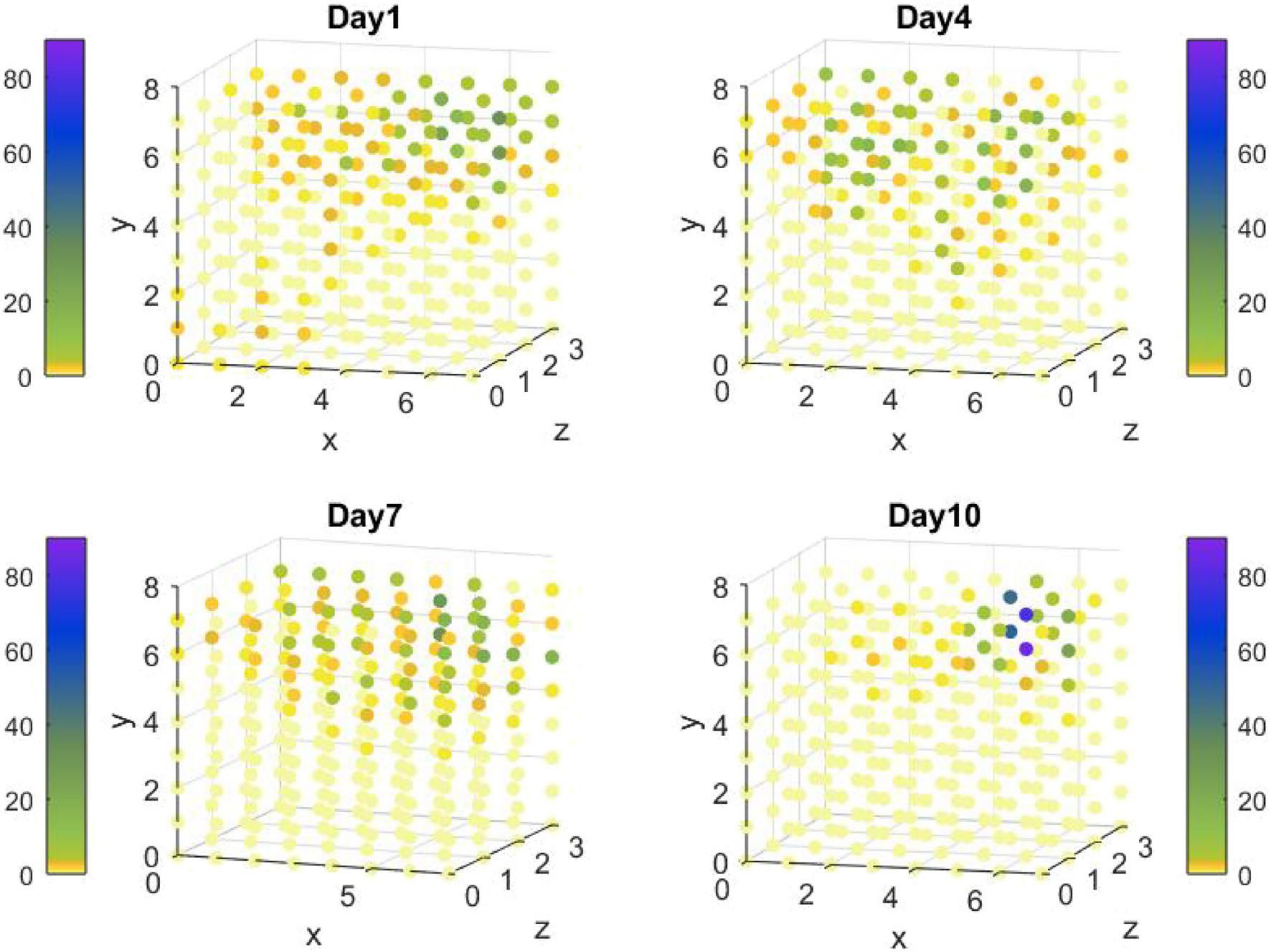
The number of bubbles appearing in the VR space on days 1,4,7 and 10 for test case 2.

Figure 5b shows the number of bubbles displayed in the virtual space on days 1, 4, 7, and 10 for test case 2. The figure highlights the dispersion of the bubbles on days 1, 4 and 7; on the first day, the algorithm has little knowledge of the patient’s capabilities and spreads the bubbles in all zones. On day 4, Mary’s physical condition changes significantly resulting in new information and dispersion of the bubbles in zone 3. From day 7 to 10 the algorithm focuses on zones 1 and 2 since Mary can pop again bubbles in these zones, so that on day 10 a larger number of bubbles is displayed in the right upper far corner of the space (Fig. 7).

The same trends are observed in Fig. 5b, describing the number of bubbles divided by zone size in the different zones along 10 days of training. Contrarily to test case 1 where the bubbles/zone size in zone 3 are extremely low (Fig. 5a), in this case the relative number of bubbles increases in zone 3 on days 4–6 following case scenario. The relative number of bubbles in zones 1 and 2 decreases abruptly but is still higher than the relative bubble number in zone 3. It is reasonable to assume that if Mary’s low kinematic measures in zones 1 and 2 were to last in the next sessions (after day 6) the relative number of bubbles in zone 3 would eventually exceed the ones in zones 1 and 2. In this case, the increase in bubbles/zone size in zone 2 is restored as soon as Mary’s physical condition improves back to its initial state.

**Figure 8:**
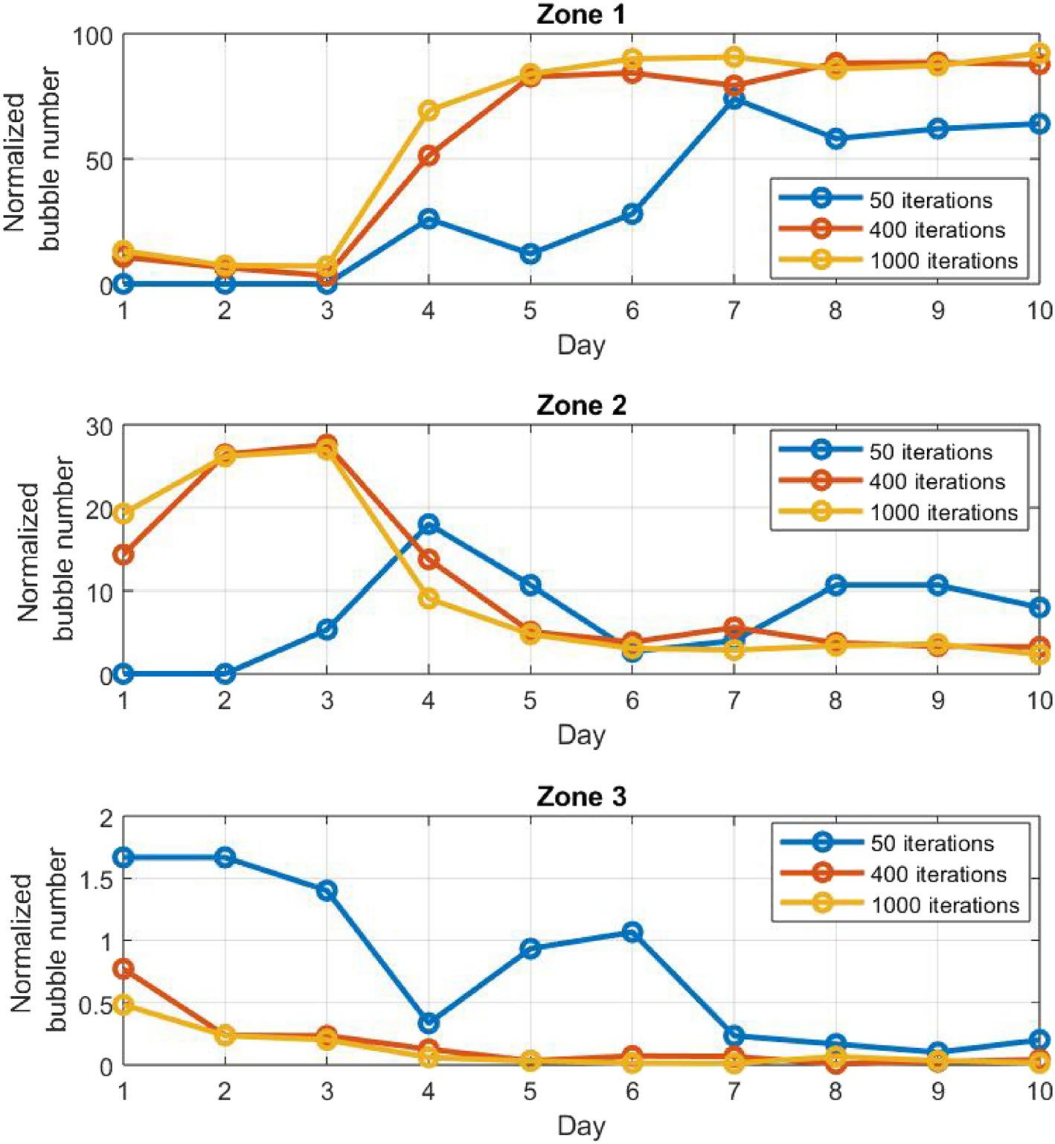
The normalized number of bubbles for each zone and day in test case 1 for 50, 400 and 1000 daily iterations.

The algorithm learns the patient’s abilities by calculating the reward and updating the Q-table at each iteration. As the bubbles cover a space of 8 × 8 × 4 = 256, the algorithm will need many iterations to learn the patient. We therefore compared the performance of the algorithm with 50, 400 and 1000 daily iterations. The results for 400 iterations are described in detail in the sections above. The comparative study is shown in Fig. 8, describing for each zone the normalized number of bubbles for different daily iteration values in test case 1. In order to compare appropriately the results for different iterations, the number of bubbles is normalized by both zone size and the number of iterations multiplied by the nominal number of iterations (400):

6 $$Normalized\,\, Bubble\, \,Number\left( {iterations} \right) = \frac{Number\, \,of\, \,Bubbles\, \,in\,\, zone}{{zone\,\, size}} \times \frac{400}{{iterations}}$$

The normalized number of bubbles is very similar for 400 and 1000 iterations in all zones. In addition, both trends show fast and similar convergence when adapting to kinematic changes on days 4 and 7. The results for 50 iterations present major differences compared to those of 400 and 1000 iterations; both the initial values on day 1 and the convergence process on days 2–3, 5–6 and 8–10 differ significantly from the higher iterations cases. The algorithm with 50 iterations does not converge in zones 2, 3 to the expected bubble values on days 3 and 6, as well as in zones 1 and 3 on day 6. On day 10, after 4 days of adjustment, the algorithm manages to reach reasonable values although still quite far from the ones achieved with 400 and 1000 daily iterations. We may then conclude that the number of iterations is a pivotal factor to consider in assessing the quality of the simulation.

## Discussion

Upper extremity function plays an important role in one’s ability to perform activities of daily living (ADL) and the loss of functionality in the use of the upper limb is a predictor of quality of life. Therefore it is essential to implement treatments aimed at its recovery.

This project focuses on the rehabilitation of reaching movement which is involved in many ADL. Reaching involves mainly the use of the proximal joint functions (shoulder and elbow). The wrist, elbow and shoulder joints work together to take the hand through space towards various targets. As every patient has specific disabilities evolving with time and training, different practice strategies are needed. An approach for a personalized and adaptive rehabilitation VR game is proposed. In this proof-of-concept study the simulation of two test cases is performed to answer the following questions.

### Does the algorithm spread the bubbles according to the treatment strategy and patient’s motor abilities?

The validity of the approach, integrating Q-learning algorithm, is explored by implementing it in two simulative test cases presenting different kinematics characteristics. From the results shown in Figs. 4, 6 and Tables 3, 4, the number of bubbles is adaptive for each zone and case, demonstrating the strength of the suggested algorithm. The number of bubbles is displayed in each zone according to the reward function expected for the different case dynamics.

The total kinematic score in each case is defined in the algorithm as an average of 4 kinematic scores representing features such as smoothness and maximal speed, previously described in movement control strategies^44,45^. Kinematic characteristics in future research may address other/additional features, as well as different formulas for the total kinematic score. Thus, this formula may be controlled according to the therapist rehabilitation protocol; for example, these kinematic characteristics may be multiplied by a weight factor. Such changes are easy to implement thanks to this simple and yet efficient algorithm.

### Does the algorithm adapt to changes in patient’s performance throughout the treatment?

In a rehabilitation process, the program is generally customized to the unique needs of the individual patient, thereby defining specific objectives and timetable. The implementation of the rehabilitation plan must respond to changes in the rate of progress and eventual medical changes. Thus, adjustments according to the patient's condition are required^2,3^. The course of the therapeutic simulation program is divided into 3 periods of time with different constant kinematic performance characteristics in each zone. In both simulated cases the kinematic characteristics are set to represent a decrease, increase or no-change in value, relative to the previous period. The results show that the algorithm adapts to these kinematic changes; moreover, the algorithm is highly responsive to kinematic features’ variation. The adaptation is achieved immediately after the change, resulting in significant fluctuations in bubble number and distribution already within the first day of change (days 1, 4 and 7). In most real scenarios, the kinematic characteristics of the patient are assumed to change more moderately, so that the algorithm’s high responsiveness will result in its faster convergence to the adequate bubble dispersion.

### How many daily iterations are needed for the algorithm to learn the patients’ abilities?

For the reinforcement algorithm to learn the environment, it must explore the different states. In the proposed approach, patient’s motoric abilities are modelled as the environment. For this to be feasible, it is necessary to perform a sufficient number of iterations for the algorithm to explore the different possible states while ensuring that the patient has the ability to fulfill the training.

In the test cases simulated here, the Q-table contains 8 × 8 × 4 = 256 rows (all possible bubble locations), and 4 columns (of possible actions). The consequent number of exploration possibilities is deducted and should be carefully considered when determining the number of iterations.

Bubble dispersion as a function of session number (day) is compared in test case 1 for different number of daily iterations (see Fig. 8); for 400 and 1000 iterations, the algorithm’s convergence rate, representing the algorithm’s ability to respond to spatial and temporal changes in user’s performance, is significantly higher than for 50 iterations. The higher the number of iterations, the higher the convergence rate, thus the more adaptive and responsive the algorithm. However, the number of iterations is also representative of the number of bubbles displayed in each session, resulting in an intrinsic conflict; in an actual rehabilitation session, a larger number of iterations means a larger number of attempted/performed movements or a longer training time. For example, if each bubble is present in the space for approximately 3 s, a number of 1000 iterations equivalent to 1000 bubbles results in 50 min of training. This is not physically feasible and even probably detrimental to patient’s condition, engagement level and recovery process. Therefore, the number of iterations is a fundamental parameter to consider, as it affects significantly the efficiency of both the algorithm and the therapeutic process. An optimization of the number of bubbles/iterations is required in future work, considering therapist’s specific recommendations regarding training time. The number of iterations may also be adaptive and vary from one session to another, according to the gradual change in patient’s condition.

### How are the reward function and algorithm parameters defined?

In Reinforcement Learning the reward function is an incentive mechanism that tells the agent what is correct and what is wrong, using reward and punishment. To benefit from RL in healthcare it is crucial to choose a correct reward function^46^. This selection is somewhat complex, as it requires expertise and it needs to balance short- and long-term reward. In the case studies in this simulation, a reward function is defined, such that gives a high reward in areas where the patient has difficulties, a smaller reward in areas that he fails to reach and a negative reward for areas where he has no difficulties. It is important to note that in future implementations an interface can be created to allow a physiotherapist or occupational therapist to define different rewards, across days and across types of disabilities. For example, for some types of injuries it may be harmful to practice at areas that cannot be reached. In this case a negative reward may be defined when a bubble is not popped. In addition, the system may be adapted to the protocols required for functional reach tests (FRT)^47^.

Other parameters may also be defined in the future by a therapist. For example, the learning rate may be determined, according to the expected rate of recovery. Another parameter is the Exploration rate $\varepsilon$, which determines how many choices will be random and how many greedy. An epsilon-greedy approach allows to balance exploitation of maximal reward and exploration by greedy choices. A dynamically decreasing exploration rate starts with a higher rate of exploration and decreases according to the number of iterations and the accumulated reward. Different values in Eq. (5) and different strategies to balance exploitation and exploration can be applied according to prior knowledge of the patient’s abilities. For example, if a more dynamic recovery is expected we may wish for a higher exploration rate, while if we expect very stable abilities a strategy with more exploitation may be more efficient.

In summary, the flexibility of the framework, showing in many configurable parameters, implies the need to pay special attention to its accessibility to physicians in embedding a personalized therapeutic policy; the implementation of the game in clinical trials should include an intuitive, user-friendly graphic user interface, with a practical description of the game’s features, monitoring of patient’s medical information, current range of motion, recommended number and duration of training sessions, performance, as well as customized parameters relevant to the algorithm as mentioned above. The model-free reinforcement learning algorithm and its independence of specific medical conditions simplifies the translation of its variables into practical multiple-choice GUI instructions, but the kinematic parameters (*m1-m4*), measuring performance, should be discussed with professionals and tailored according to pathology and therapeutic strategy. To this end, a bank of documented kinematic parameters and a customizable scale of importance (determining the weight of parameters *m1–m4* in Eq. (3)) may be added.

### What are the limitations of the framework?

In implementing the proposed framework in real clinical trials, the validity and accuracy of the patient’s kinematic parameters, based on the measurement of hand position, should be considered. Several works discuss the accuracy and reliability of head-mounted displays and controllers in tracking translational and rotational movement, such as HTC VIVE, Oculus Rift S, Oculus Touch and Meta Quest 2^48–51^. The systems are found to be suitable for biomechanical and motor rehabilitation applications. However, the heterogeneity of the findings suggests that specific setups, including tracking space size and distance between measurement points influences the positional error^50^. Carnevale et al.^52^ evaluated the accuracy of the Oculus Quest 2 (Meta Quest 2) VR system compared to a Qualisys optical capture system in measuring translational and rotational displacements for shoulder rehabilitation applications. In a translational range of 200 to 700 mm (corresponding to anthropometric forearm and upper limb evaluations), they reported a mean absolute error of 13.52 ± 6.57 mm at 500 mm from the HMD in the x-direction. The maximum mean absolute error for rotational displacements was found to be 1.11 ± 0.37° for a rotation of 40° around the z-axis. In a different setup, Abdlkarim et al. reported an average positional error of the fingertip of 11 mm, an average finger joint angle error of 9.6° and an average temporal delay of 38 ms^51^. Extrapolating the results in^52^ to the proposed VR game in our work with the current bubble spatial distribution (Fig. 3), a displacement error of 2.2% in the VR game space may correspond to 2.75 mm error in bubble position precision, depending on space size definition. Moreover, these studies do not consider variations in movement’s velocity; increasing movement speed will affect (increase) positional error. Therefore, a setup-specific analysis and evaluation of spatial and temporal accuracy will be imperative in determining bubble distribution resolution in the 3D virtual space before clinical trials.

Although immersive VR-based interventions are shown in literature to be beneficial in motor and neurorehabilitation, larger scale clinical studies are needed, including a systematic validation of VR tracking devices.

The resolution of bubble distribution space influences the number of states in the 3D space that implicitly changes the number and/or duration of iterations/sessions needed. Because of the nature and number of possible actions (Up, Down, Right, Left, Forward, Backward) dictated by the space, the basic Q-learning algorithm proposed in this work manages to optimize bubble location with relatively few iterations, without the need of offline simulation models (as opposed to^35^). However, the present bubble distribution, related to the number of states in space, requires optimization and validation in real rehabilitation interventions, ensuring a balance between training time and algorithm performance, as mentioned earlier.

## Conclusions

Virtual Reality has a great potential in extending rehabilitation therapy in the clinic and at home. For the alternative interventions to be effective, adaptive and personalized rehabilitation programs are much needed; they may introduce a new generation of engaging, flexible home-training with immediate feedback, adaptive response and monitoring. This study proposes the implementation of Reinforcement Learning, and specifically Q-learning in the customization of a rehabilitation serious game conceived for reaching movement treatment. The game presents in a virtual space bubbles to be reached and popped by the user, and adjusts in real time the number of bubbles in space according to therapeutic strategy and patient’s personal temporal and spatial abilities. The algorithm is validated by the simulation of two different test cases, presenting changing kinetic characteristics in time. The game presents more bubbles in the zones where a higher reward is defined and shows fast adaptive response to the changes in the patient’s ability throughout days of practice. The simulation suggests that the algorithm offers good adaptive capabilities, and its relative simplicity enables further implementation of a user interface that will provide the therapist with the possibility to adapt the program’s parameters to each individual therapeutic strategy. Moreover, the therapist will be able to control the timing of bubbles’ appearance/disappearance as well as the overall training time, thereby determining the total number of bubbles and attempted movements per training. Future work will demonstrate the concept by clinical trials, where the kinematic characteristics of the patient will be measured and analyzed in real time to provide the algorithm with the data currently supplied by the simulation of test cases.

## Data Availability

The datasets generated and/or analysed during the current study, including the simulation code and simulation matrices, are available in the GitHub repository at https://github.com/anatdhn/RLSimulaton/tree/main.

## Abbreviations

VR: Virtual reality
ADL: Activities of daily living
HMD: Head-mounted display
AI: Artificial intelligence
ROM: Range of motion
FRT: Functional reach test
